# Variation in Cannabinoid Metabolites Present in the Urine of Adults Using Medical Cannabis Products in Massachusetts

**DOI:** 10.1001/jamanetworkopen.2021.5490

**Published:** 2021-04-12

**Authors:** Jodi M. Gilman, William A. Schmitt, Grace Wheeler, Randi M. Schuster, Jost Klawitter, Cristina Sempio, A. Eden Evins

**Affiliations:** 1 Center for Addiction Medicine, Department of Psychiatry, Massachusetts General Hospital, Boston; 2 Harvard Medical School, Boston, Massachusetts; 3 Athinoula A. Martinos Center for Biomedical Imaging, Charlestown, Massachusetts; 4 Department of Anesthesiology, University of Colorado, Aurora

## Abstract

This cohort study examines the association between medical cannabis product use and exposure to Δ9-tetrahydrocannabinol and cannabidiol by quantifying levels of their metabolites in urine.

## Introduction

A growing market of medical cannabis products claim to have specific Δ9-tetrahydrocannabinol (THC) and cannabidiol (CBD) content, but regulation of THC and CBD content is inconsistent across states and generally weak.^1^ To examine the association between medical cannabis product use and exposure to THC and CBD, we quantified levels of THC, CBD, and their metabolites in urine of participants in a clinical trial (ClinicalTrials.gov identifier NCT03224468) of medical cannabis in Massachusetts.

## Methods

This cohort study was approved by the Partners Human Research Committee. All participants provided written informed consent. This study follows the Strengthening the Reporting of Observational Studies in Epidemiology (STROBE) reporting guideline.

Adults (aged 18-65 years) with a desire to use cannabis for depression, pain, or insomnia were recruited through advertising and were assessed at baseline and 2, 4, 12, 24, and 48 weeks after initiating cannabis. The study took place between June 2017 and August 2020. At each visit, participants provided a urine sample, reported recency of cannabis use, and reported whether their primary products were THC-dominant, CBD-dominant, or approximately equal CBD and THC. Samples collected during visits in which participants reported using products from licensed dispensaries within the prior 48 hours and at least 3 to 4 days per week since the previous visit were analyzed using high-performance liquid chromatography with tandem mass spectrometry.^2^

We ran separate models for THC and CBD metabolites using Kendall rank correlations for ordinal variables and logistic regressions for nominal variables, using R statistical software version 4.0 (R Project for Statistical Computing). All tests and 95% CIs were 2-sided, and significance was defined as *P* < .05. For logistic regressions, we performed a Wald test. Data analysis was performed from September 2020 to February 2021.

## Results

Ninety-seven participants (mean [SD] age, 39.6 [14.74] years; 65 women [67.01%]) provided 256 urine samples meeting the criteria for analysis. Participants were light users at baseline (53% used less than monthly). After baseline, 39% to 47% used 3 to 4 days per week, 15% to 20% used 5 to 6 days per week, and 29% to 45% used daily.

At least 1 cannabis metabolite was detected in 220 samples (85.9%) (Table 1). Among participants who reported using CBD-dominant or equal CBD-THC products, there was no detectable CBD metabolite in 10 samples (30.3%) and 20 samples (37.0%), respectively (Table 2). THC was detected in 26 samples (78.8%) from participants reporting use of CBD-dominant products. Among samples from participants reporting THC-dominant or equal CBD-THC products, no THC metabolites were present in 13 samples (10.9%) and 19 samples (35.2%), respectively.

**Table 1.** Cannabinoid Metabolite Measurements in 256 Urine Samples From 97 Participants

Metabolite	Limit of quantitation, ng/mL	Samples with detectable metabolite, No. (%)	Concentration, median (range), ng/mL^a^^,^^b^
THC metabolites			
THC-COO-Gluc	7.8-2000	209 (81.6)	40.6 (<7.8-22353.6)
THC-COOH	0.39-400	136 (53.1)	0.5 (<0.39-407.6)
THC-Gluc	0.78-200	64 (25.0)	<0.78 (<0.78-182.2)
THCV-COOH	0.78-400	25 (11.1)	<0.78 (<0.78-43.5)
11-OH-THC	1.56-400	10 (3.9)	<1.56 (<1.56-3.9)
THC	0.78-400	0	NA
THCV	0.78-400	0	NA
Any THC metabolite^c^	NA	209 (81.6)	NA
CBD metabolites			
CBD-Gluc	0.78-100	116 (45.3)	<0.78 (<0.78-215.6)
7-CBD-COOH	0.78-400	43 (16.8)	<0.78 (<0.78-28.8)
7-OH-CBD	3.13-400	28 (10.9)	<3.13 (<3.13-44.6)
6α-OH-CBD	1.56-400	10 (3.9)	<1.56 (<0.78-9)
6βb-OH-CBD	0.78-400	9 (3.5)	<0.78 (<0.78-7.4)
CBD	0.78-400	1 (0.4)	<0.78 (<0.78-1.1)
CBDV	0.39-400	0	NA
Any CBD metabolite^d^	NA	142 (55.5)	NA
Other metabolites			
CBC	1.56-400	1 (0.4)	<1.56 (<1.56-0.9)
CBG	0.39-400	0	NA
CBN	0.78-400	0	NA
Any cannabinoid metabolite (sum)	NA	220 (85.9)	NA
No cannabinoid metabolites detected	NA	36 (14.0)	NA

Abbreviations: 11-OH-THC, 11-hydroxy-Δ9-tetrahydrocannabinol; 6α-OH-CBD, 6-alpha-hydroxy-cannabidiol; 6β-OH-CBD, 6-beta-hydroxy-cannabidiol; 7-OH-CBD, 7-hydroxy-cannabidiol; 7-CBD-COOH, (3R-trans)-cannabidiol-7-oic acid; CBC, cannabichromene; CBD, cannabidiol; CBD-Gluc, cannabidiol glucuronide; CBDV, cannabidivarin; CBG, cannabigerol; CBN, cannabinol; NA, not applicable; THC-COO-Gluc, 1-nor-Δ9-tetrahydrocannabinol-9-carboxylic acid glucuronide; THC-COOH, 11-Nor-9-carboxy- Δ9-tetrahydrocannabinol; THCV-COOH, 11-nor-9-carboxy-Δ9-tetrahydrocannabivarin; THC-Gluc, Δ9-tetrahydrocannabinol glucuronide; THCV, Δ9-tetrahydrocannabivarin.

^a^ When calculating the median, concentrations falling below the lower limit of quantification were imputed as the lower limit of quantification.

^b^ Values above the upper limit of quantification are estimates.

^c^ In total, 72% of participants had detectable THC metabolites at all visits, and 9% had undetectable THC metabolites at all visits; 19% of participants were variable, with detectable THC metabolites at some visits but not others. There was no association between frequency of use and presence of THC metabolites (Kendall τ = 0.09; *P* = .09).

^d^ In total, 22% of participants had detectable CBD metabolites at all visits, and 19% of participants had undetectable CBD metabolites at all visits; 59% of participants were variable, with detectable CBD metabolites at some visits but not others. There was an association between frequency of use and presence of CBD metabolites (Kendall τ = 0.21; *P* < .001). THCV-COOH only has values for 224 samples because of a lack of reference THCV-COOH in 1 batch.

**Table 2.** Presence of CBD or THC Metabolites by Self-reported CBD-THC Content and Primary Route of Administration

Self-report category	Total samples, No.	Metabolite detected, samples, No. (%)				
		CBD		THC		Neither
		Yes	No.	Yes	No	
All product types						
CBD dominant	33	23 (69.7)	10 (30.3)	26 (78.8)	7 (21.2)	5 (15.2)
Equal CBD and THC	54	34 (63.0)	20 (37.0)	35 (64.8)	19 (35.2)	13 (24.1)
THC dominant	119	63 (52.9)	56 (47.1)	106 (89.1)	13 (10.9)	11 (9.2)
Not sure or data not provided	50	22 (44.0)	28 (56.0)	42 (84.0)	8 (16.0)	7 (14.0)
By route of administration						
Vaped^a^	137	67 (48.9)	70 (51.1)	101 (73.7)	36 (26.3)	27 (19.7)
CBD dominant	11	9 (81.8)	2 (18.2)	10 (90.9)	1 (9.1)	1 (9.1)
Equal CBD and THC	40	24 (60.0)	16 (40.0)	23 (57.5)	17 (42.5)	11 (27.5)
THC dominant	58	25 (43.1)	33 (56.9)	47 (81.0)	11 (19.0)	9 (15.5)
Not sure or data not provided	28	9 (32.1)	19 (67.9)	21 (75.0)	7 (25.0)	6 (21.4)
Oral^b^	66	49 (74.2)	17 (25.8)	60 (90.9)	6 (9.1)	4 (6.1)
CBD dominant	20	14 (70.0)	6 (30.0)	15 (75.0)	5 (25.0)	3 (15.0)
Equal CBD and THC	10	9 (90.0)	1 (10.0)	10 (100.0)	0	0
THC dominant	28	19 (67.9)	9 (32.1)	27 (96.4)	1 (3.6)	1 (3.6)
Not sure or data not provided	8	7 (87.5)	1 (12.5)	8 (100.0)	0	0
Smoked^c^	53	26 (49.1)	27 (50.9)	48 (90.6)	5 (9.4)	5 (9.4)
CBD dominant	2	0	2 (100.0)	1 (50.0)	1 (50.0)	1 (50.0)
Equal CBD and THC	4	1 (25.0)	3 (75.0)	2 (50.0)	2 (50.0)	2 (50.0)
THC dominant	33	19 (57.6)	14 (42.4)	32 (97.0)	1 (3.0)	1 (3.0)
Not sure or data not provided	14	6 (42.9)	8 (57.1)	13 (92.9)	1 (7.1)	1 (7.1)

Abbreviations: CBD, cannabidiol; THC, Δ9-tetrahydrocannabinol.

^a^ The most commonly reported measure of dose was in “hits” (134 hits; mean, 3.2 hits; range, 1-25 hits). For vaped cannabis measured in hits, there was no association between the presence of CBD metabolites and dosage (Kendall τ = 0.07; *P* = .36) or the presence of THC metabolites and dosage (Kendall τ = 0.03; *P* = .71).

^b^ The most commonly reported measure of dose was in milligrams (40 doses; mean, 8.4 mg; 2-25 mg) or drops (8 doses; mean, 4.4 drops; range, 1-10 drops). For oral cannabis measured in milligrams, there was no association between presence of CBD metabolites and dosage (Kendall τ = 0.01; *P* = .90), but a significant association between the presence of THC metabolites and dosage (Kendall τ = −0.36; *P* = .01).

^c^ The most commonly reported measure of dose was in hits (39 hits; mean, 4.9 hits; range, 1-15 hits) or joints (7 joints; mean, 1.2 joints; range, 0.5-3.5 joints). For smoked cannabis measured in hits, there was an association between the presence of CBD metabolites and dosage (Kendall τ = 0.44; *P* = .001), as well as the presence of THC metabolites and dosage (Kendall τ = 0.40; *P* = .004).

Although vaping was the most common method of administration, 27 samples (19.7%) from participants who reported vaping contained no measurable cannabinoid whatsoever. CBD metabolites were more likely to be detected in participants who used oral than vaped (odds ratio [OR], 3.01; 95% CI, 1.58-5.74; *P* < .001) or smoked (OR, 2.99; 95% CI, 1.38-6.47; *P* = .005) products. THC metabolites were more likely to be detected in participants who used oral (OR, 3.56; 95% CI, 1.42-8.96; *P* = .007) and smoked (OR, 3.42; 95% CI, 1.26-9.27; *P* = .02) products than in vaped products.

## Discussion

Among adults using medical cannabis frequently and recently, THC and CBD metabolite concentrations in urine often differed from expected exposure. Approximately one-third of samples from people reporting using CBD-dominant products contained no measurable CBD metabolite. Nearly 1 in 5 samples from those using vaped cannabis contained no detectable cannabinoids. There were no dose-metabolite associations for vaped products. This may indicate that vaping devices may not heat cannabis products appropriately, and US Food and Drug Administration–approved devices may deliver more consistent cannabinoid exposure. Product and delivery method variability present challenges to assessing the efficacy and safety of medical cannabis.

Methodological limitations of this study include participant-determined doses, possible errors in self-report, no analysis of the cannabis products themselves, and individual differences in rate of absorption and metabolism. Products were purchased in Greater Boston dispensaries, so results may not generalize to regions with different regulations.

The findings of this cohort study are consistent with those of a study^1^ of cannabis products purchased in California and Washington, in which more than one-half of products were incorrectly labeled. These findings indicate that adults using medical cannabis products may have incomplete or incorrect information regarding expected cannabinoid exposure from these purchased products, impeding informed patient choice and investigation of pharmacologic and therapeutic properties of cannabis products.
